# A Rare Sparkle: A Case of Calcified Kidneys in a Young Infant With Renal Failure

**DOI:** 10.7759/cureus.46827

**Published:** 2023-10-11

**Authors:** Aliza Mittal, Hritvik Jain, Amarpal Singh, Taruna Yadav, Vikarn Vishwajeet

**Affiliations:** 1 Department of Pediatrics, All India Institute of Medical Sciences, Jodhpur, Jodhpur, IND; 2 Department of Diagnostic and Interventional Radiology, All India Institute of Medical Sciences, Jodhpur, Jodhpur, IND; 3 Department of Pathology, All India Institute of Medical Sciences, Jodhpur, Jodhpur, IND

**Keywords:** oxalate crystals, hyperoxaluria, oxalosis, rapidly progressive renal failure, primary hyperoxaluria type 1, primary hyperoxaluria

## Abstract

Primary hyperoxaluria-1 (PH1) is an autosomal recessively inherited rare genetic condition due to the deficiency of the hepatic enzyme alanine:glyoxylate aminotransferase which leads to high systemic levels of oxalate and subsequently, early end-stage renal disease and death. Here, we present a case of a three-month-old male infant who presented with loose stools, reduced oral intake, and decreased activity for 12-13 days along with edema and a peeling rash on cheeks, lips, and genitalia. During the entire duration of the inpatient stay, the child was oligoanuric. Kidney ultrasound (USG) was suggestive of bilateral hyperechoic kidneys with increased cortical echogenicity and a computed tomography scan showed bilateral diffusely calcified renal cortices with well-preserved renal architecture. A diagnosis of “oxalate nephropathy” was made from renal biopsy and genetic testing confirmed it to be “primary hyperoxaluria-1”. The child was initially managed conservatively, and then peritoneal dialysis was done, following which the child was shifted to intermittent hemodialysis.

## Introduction

Primary hyperoxaluria-1 (PH1) is an autosomal recessive disorder caused by an enzyme deficiency of alanine:glyoxylate-aminotransferase (AGT) in the liver peroxisomes and has a prevalence of 1-3 cases per 1 million [1]. The three most common mutations accounting for 34.5% of cases are c.33_34insC, c.508G>A, and c.731T > C [2]. Owing to its rarity, PH1 is often misdiagnosed or diagnosed in later stages [3]. AGT is a pyridoxal-dependent enzyme that catalyzes the conversion of glyoxylate to glycine, a deficiency of which leads to the conversion of glyoxylate to oxalate. As end-stage renal disease (ESRD) sets in, systemic oxalate clearance decreases due to a decline in renal function, leading to elevated oxalate levels, known as oxalosis (plasma oxalate >30-50 μmol/L [3 = Cochat P 2012]). Hyperoxaluria leads to the formation of calcium oxalate urinary crystals in supersaturated urine that leads to urolithiasis or nephrocalcinosis. Patients eventually develop ESRD because of interstitial fibrosis and renal parenchymal inflammation brought on by urolithiasis and nephrocalcinosis [4]. We hereby report a case of a three-month-old male baby with PH1. 

## Case presentation

A three-month-old male infant presented with loose stools, reduced oral intake, and decreased activity for 12-13 days. The child was born out of a non-consanguineous marriage, to a G4P3A0L2 (Gravida; Para; Abortions; Live Births) mother by caesarean section (in view of pre-eclampsia). There was a history of previously unexplained sibling death in the early neonatal period. His birth weight was 3 kg and no history of neonatal complications, jaundice, or neonatal intensive care unit (NICU) stay at birth. The antenatal period was uneventful; no other significant family history was noted. With the present complaints, no blood or mucus was associated with the loose stools. There was no history of fever, vomiting, abdominal distension, cough, bleeding manifestations, drug intake, or decreased urine output. Pre-morbidly, the child had been partially immunized (only at birth). His developmental milestones were normal. 

With this presentation, he initially presented to an outside hospital, where he received intravenous antibiotics (undocumented) and supportive treatment. The supportive treatment included maintenance of adequate hydration, oxygen therapy, maintenance of blood sugars, and maintaining normal body temperature. At admission to that hospital, the child was unconscious and afebrile and had pallor along with mild pedal and periorbital edema. Vitally, the child had tachycardia (122 bpm), tachypnea (52 bpm), and hypertension (110/70 mmHg; >95th percentile). On examination, the child was irritable with a Glasgow Come Score (GCS) of 12/15 and had respiratory distress with nasal flaring and fine bilateral basilar crepitations. On day 4 of the hospital stay, he developed oliguric acute kidney injury (AKI) with fluid overload with metabolic acidosis for which peritoneal dialysis was initiated and was performed for 72 hours. However, AKI had not improved. On day 10, the child developed multiple bullous lesions all over the body with a positive Nikolsky sign and had pallor along with anasarca. A working diagnosis of “clinical sepsis with AKI with Steven Johnson Syndrome” was made. 

Given multiple morbidities (oliguria, post-peritoneal dialysis status, Steven Johnson syndrome, and septicemia), the child was referred to us at a tertiary care center. On admission to the pediatric intensive care unit, the child was unconscious. Vitally, the child had tachycardia (142/min), hypertension (112/70), and tachypnoea (66/min), with a SpO_2_ of 96% on a high-flow nasal cannula. On head-to-toe examination, the child had pallor, edema, and rash. The anterior fontanelle (AF) was 2.5 cm and non-bulging. Peeling of skin in the occipital region was noted. Periorbital edema with an erythematous rash on cheeks, lips, genitalia, and oral mucosa was seen, also the rash was positive for Nikolsky sign.

The patient was continued on intravenous antibiotics and intravenous immunoglobulin was administered for bullous skin lesions, alongside other supportive measures. The respiratory distress was supported by mechanical ventilation. The child remained oliguric with a urine output of only 0.5 ml/kg/hr. By day 14, the baby had fluid overload (18%) (weight = 5.5 kg), and a peritoneal catheter was inserted again at a different site to restart peritoneal dialysis considering the small size of the child. However, the baby developed frank bleeding from the peritoneal catheter, the peritoneal dialysis was stopped, and the child was then shifted to continuous renal replacement therapy (CRRT). 

To evaluate the cause of peritoneal catheter bleeding, a non-contrast computed tomography abdomen (NCCT) was performed. It was found that a muscular artery lying in the course of the peritoneal catheter was severed. Conservative management included no active medical or surgical intervention but allowing the bleed to stop on its own by tamponade effect helped stop this bleed. Meanwhile, the fluid overload was successfully managed by CRRT which was continued for 48 hours. Respiratory support was gradually weaned off as the fluid overload improved with ultrafiltration and he was eventually stable on oxygen via nasal prongs. 

**Figure 1:**
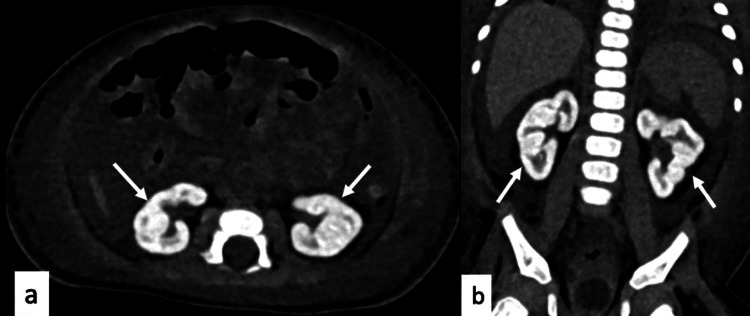
NCCT Abdomen showing bilateral diffuse calcified kidneys (white arrows) with well-preserved renal architecture. (a) Axial view and (b) coronal view.

During the entire inpatient stay, the child was oligoanuric. The child was later shifted to intermittent hemodialysis for fluid removal. Kidney ultrasound was suggestive of bilateral hyperechoic kidneys with increased cortical echogenicity. The NCCT abdomen earlier performed showed bilateral diffusely calcified renal cortices with well-preserved renal architecture (Figure 1). 

**Figure 2:**
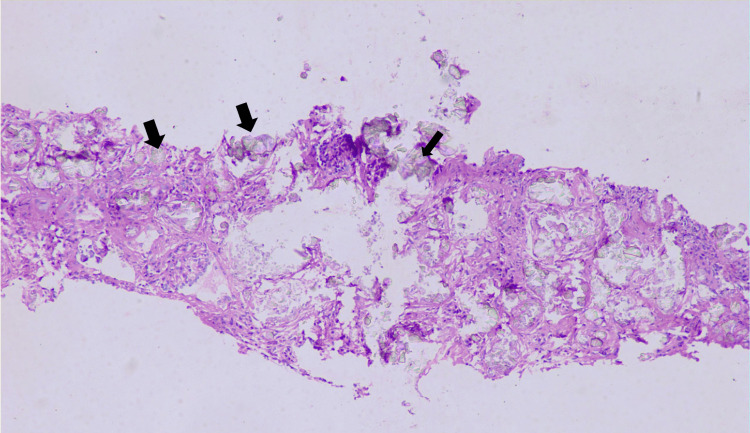
Biopsy shows the presence of refractile crystals in the renal tubules (black arrows). The renal tissue was stained using Hematoxylin and Eosin (H&E) stain and was viewed under 40x magnification.

**Figure 3:**
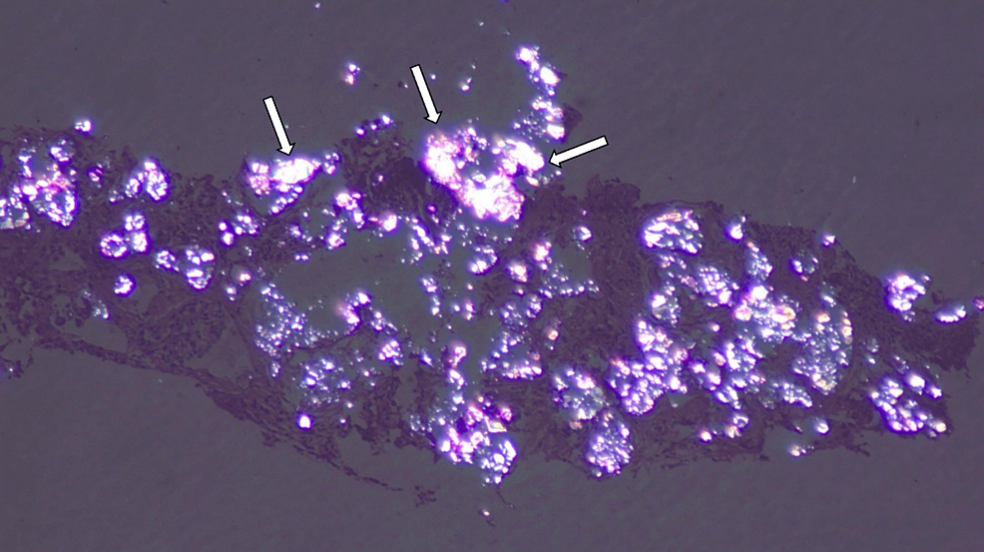
Biopsy on polarising microscopy appears birefringent (white arrows) suggesting crystalline nephropathy.

This finding of calcified kidneys led to the suspicion of an underlying monogenic or metabolic disorder. In the absence of iatrogenic causes, the other preliminary investigations helped in ruling out pathological causes of infantile nephrocalcinosis like Bartter syndrome, Dent disease, hyperuricosuria, cystinuria, and tyrosinemia. Urine analysis confirmed the presence of oxalate crystals along with red blood cell casts and proteinuria. A renal biopsy was performed for prognosis and diagnosis, which showed crystals in the renal tubules (Figure 2) that appeared birefringent on polarizing microscopy (Figure 3) and the biopsy was suggestive of crystalline nephropathy. 

In the absence of any drug causing crystalline nephropathy, a small baby with early-onset AKI progressing to renal failure, the most likely plausible explanation was “oxalate nephropathy”. Clinical exome genetic testing in this case revealed a likely pathogenic, homozygous missense variation in the exon 2 of the AGXT gene [AGXT +, Exon 2 c.302T>C(p.Leu101Pro)Homozygous], confirming PH1. 

The child was anuric until he was discharged from the hospital as the child's family was not willing to continue dialysis or a combined liver-kidney transplantation; hence, they opted for palliative care. The parents of the child were informed of genetic counseling for future pregnancies. Unfortunately, two weeks later, the child succumbed to death at his home.

## Discussion

PH1 should be suspected as the underlying disorder in a child with a first kidney stone or in an adult with recurrent kidney stones, an infant with failure to thrive along with impaired renal function markers, in a case of unexplained renal failure (particularly in the presence of nephrocalcinosis), presence of refractile, birefringent oxalate crystals in any bodily fluid, severe urolithiasis, and a positive family history of PH1 [5,6]. The infantile form of PH1 is known to be life-threatening particularly due to the rapid progression to ESRD and the mortality rate of infantile PH1 has been reported to be nearly 100% as opposed to the 14.3% mortality rate in non-infantile PH1 [7]. 

On ultrasound, the kidneys show diffuse or medullary echogenicity in the early disease but may have cortical echogenicity in ESRD. In this case presentation, it was surprising to see complete calcification of the entire renal cortex early in the disease. Twenty-four-hour urinary oxalate level >0.5 mmol/1.73m^2^/day or spot urine oxalate creatinine ratio above the reference range in the absence of secondary causes usually favors the diagnosis. A plasma oxalate >100 μmol/L is suggestive of primary hyperoxaluria, but it may not be available in most settings and is not useful until the glomerular filtration rate declines below 30 ml/min/1.73m^2^. 

In a retrospective analysis of PH1 cases by Lin et al., the median age of symptom onset was 12 months (IQR 3-63 months) and the median age at PH1 diagnosis was 14 months (IQR 4-136 months) [7]. In a descriptive cohort study by Soliman et al., the median age at initial symptoms was three years but at diagnosis was six years [8]. The onset of ESRD differed in various age groups, with 35.3% of patients reaching ESRD in infancy, 23.5% reaching ESRD in early childhood, and 41.29% reaching ESRD in late childhood [8]. A retrospective review by Al Riyami et al. concluded the median age at presentation to be 7 months (IQR 1-60 months) [9]. At presentation, 39% of patients were in severe renal failure, majority of them were below 1 year of age [9]. Hence, the median age of presentation and diagnosis varies widely, but literature on 3-month-olds presenting with renal failure is scarce.

The use of liver biopsy specimens for enzyme estimation has now been limited only to cases where suspicion of PH is high [1-3,10]. More than 150 mutations in the AGXT gene have been detected so far. The three most common mutations accounting for 34.5% of cases are c.33_34insC, c.508G>A, and c.731T > C. In this given patient, the mutation was c.302T>C (p. Leu 101 Pro) and has also previously been reported in a single PH1 patient [11]. Pyridoxine is known to lower urinary oxalate excretion in some specific PH1 genotypes like c.508G>A and C.454T>A by enhancing AGT activity [12]. 

Aggressive hydration and crystallization inhibitors are used for the treatment and prevention of renal damage. In advanced cases, regular dialysis is done apart from transplantation (sequential or combined liver and kidney). Recently newer treatment modalities for PH1 including RNA interference, namely, Lumasiran [13] and Nedosiran [14] have emerged which are potential game-changers for the successful treatment of PH1 as these RNAi molecules reduce the need for undergoing transplantation in PH1 [15]. With the advent of artificial intelligence (AI) and machine learning (ML) in the field of pediatrics, there are ample opportunities to ensure clinical benefits to patients by using AI-ML models [16]. ML models may be integrated with clinical decisions to identify at-risk pediatric populations, particularly for rare diseases to provide evidence-based recommendations [17]. The curation of large clinical and scientific databases will aid in the formation of accurate diagnoses and also provide clinicians with updated information [16]. Rapid exome sequencing (RES), particularly in consanguineous populations, can improve acute management changes wherein an urgent molecular diagnosis can influence the management. Monies et al. demonstrated the clinical utility of RES in a consanguineous population of 189 cases [18]. The spectrum of genetic testing should be widened with the inclusion of RES, particularly in the diagnosis of genetic disorders like PH1 as RES can influence the management of these disorders positively [18]. 

## Conclusions

This case report highlights the challenges in diagnosing and managing PH1 and the importance of early detection. The key takeaway from this case report is the pivotal role of strong clinical suspicion. Although AKI in the setting of sepsis is commonly noted in infancy, it is important to exclude inherited disorders when managing refractory oliguric or severe AKI. Calcified kidneys in early infancy are a pointer to an underlying inherited metabolic disorder like PH1. PH1 is potentially treatable due to advancements in intensive high-flux hemodialysis as well as combined liver and kidney transplantation procedures. PH1 can rapidly lead to ESRD if not treated early, and once ESRD sets in, conservative management must be replaced with advanced medical and surgical options. Awareness among clinicians is of utmost importance for rare disorders like PH1 to facilitate early diagnosis and prompt intervention. A multidisciplinary approach, with collaboration among nephrologists, geneticists, and urologists, is of paramount importance in the successful management of PH1. Early efforts to mitigate systemic oxalosis are keys to delaying the devastating consequences of PH1 and improving the outcomes of treatment. 
